# Ion agent mitigates efficiency roll-off in near-infrared electroluminescence for practical bioimaging and information encryption

**DOI:** 10.1038/s41377-026-02237-1

**Published:** 2026-05-07

**Authors:** Tao Yang, Ye Wang, Zong-Shuo Liu, Feng Zhao, Wei-Zhi Liu, Wan-Shan Shen, Ya-Kun Wang, Liang-Sheng Liao

**Affiliations:** 1 Institute of Functional Nano & Soft Materials (FUNSOM), Jiangsu Key Laboratory for Carbon-Based Functional Materials & Devices, State Key Laboratory of Bioinspired Interfacial Materials Science, Soochow University, 215123 Suzhou, Jiangsu China; 2 Macao Institute of Materials Science and Engineering, Macau University of Science and Technology, Macau SAR, 999078 Taipa, China

**Keywords:** Quantum dots, Inorganic LEDs

## Abstract

Perovskite quantum dots (PQDs) are promising emitters for next-generation light-emitting diodes (LEDs), yet PQD-based near-infrared (NIR) LEDs still suffer from low external quantum efficiencies (EQEs) and severe efficiency roll-off. This limitation arises from the trade-off between enhancing carrier transport with conductive ligands and preserving PQD surface integrity during ligand exchange. Here, we report an ionic liquid-mediated surface reconstruction strategy that simultaneously stabilizes PQD surface and enhances charge transport. Incorporating the multifunctional ionic liquid 1-methyl-3-propylimidazolium iodide (MPII) into the antisolvent suppresses defect formation while forming an in situ protective layer, effectively reducing surface traps and preserving PQD structural integrity. The treated PQD films exhibit a twofold reduction in trap density and a tenfold increase in conductivity, ensuring balanced carrier injection and efficient radiative recombination. As a result, the fabricated NIR LEDs achieve a record EQE of 24.8%, maintaining ~20% EQE at a radiance of 10 W sr^-^¹ m^-^²—representing the lowest efficiency roll-off for PQD-based NIR LEDs reported to date. Furthermore, large-area devices (900 mm²) reach EQEs of up to 20% and demonstrate practical applications in biomedical imaging and information encryption, underscoring the broad potential of this strategy for high-performance NIR optoelectronics.

## Abstract (web summary)

An ionic liquid-mediated surface reconstruction strategy is developed to mitigate the severe efficiency roll-off in PQD-based NIR LEDs, achieving a record EQE of 24.8% with significantly suppressed efficiency roll-off.

## Graphical abstract

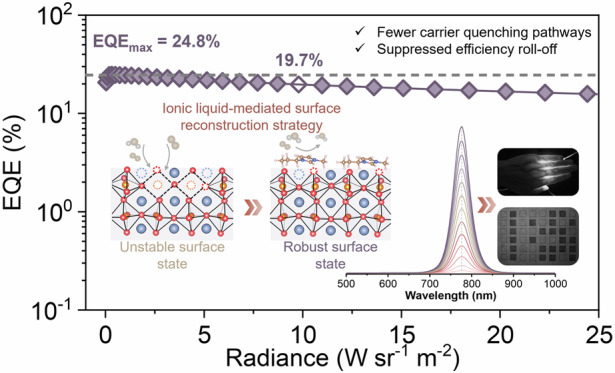

## Introduction

Light-emitting diodes (LEDs) underpin modern optoelectronics, enabling technologies from energy-efficient displays to biomedical imaging and optical communications^1–3^. Extending their operation into the near-infrared (NIR) spectral window unlocks applications such as non-invasive diagnostics, night-vision surveillance, and high-speed data transfer, where long-wavelength photons provide deeper tissue penetration and reduced scattering losses^4–7^. However, achieving efficient and stable NIR emission remains an outstanding challenge.

Perovskite quantum dots (PQDs) have emerged as highly promising emitters owing to their near-unity photoluminescence quantum yields (PLQY) and defect-tolerant electronic structures^8–15^. While PQD-based visible LEDs have rapidly reached external quantum efficiencies (EQEs) exceeding 20%^16–18^, NIR counterparts have stagnated below 20% and suffer from severe efficiency roll-off at high brightness—a critical bottleneck for practical deployment^19–22^.

The root of this limitation lies in the fragile surface chemistry of PQDs^23,24^. Conventional ligand exchange strategies—designed to replace insulating long-chain ligands with shorter conductive ones—can improve carrier transport but often destabilize the PQD lattice, induce surface traps, and trigger aggregation, particularly because these exchanges rely on polar solvents^25–27^. This intrinsic trade-off between conductivity and stability has fundamentally limited NIR device performance.

Here, we overcome this trade-off by developing an ionic liquid-mediated surface reconstruction strategy. Incorporating the multifunctional ionic liquid 1‑methyl‑3‑propylimidazolium iodide (MPII) into the antisolvent during the purification process simultaneously suppresses defect formation and forms an in situ protective layer, preserving lattice integrity while boosting film conductivity. The treated PQD films exhibit significantly improved phase stability with nearly twofold lower trap densities and an order-of-magnitude higher conductivity than control films, enabling balanced carrier injection and efficient radiative recombination. As a result, PQD-based NIR LEDs achieve a record EQE of 24.8% with the lowest efficiency roll-off reported to date: the EQE remains approximately 20% even at a high radiance of 10 W sr^-1^ m^-2^. Moreover, we demonstrate large-area (900 mm²) devices with EQEs up to 20%, together with practical applications in biomedical imaging and information encryption, highlighting the potential of this approach for advancing high-performance NIR optoelectronics.

## Results

**Fig. 1:**
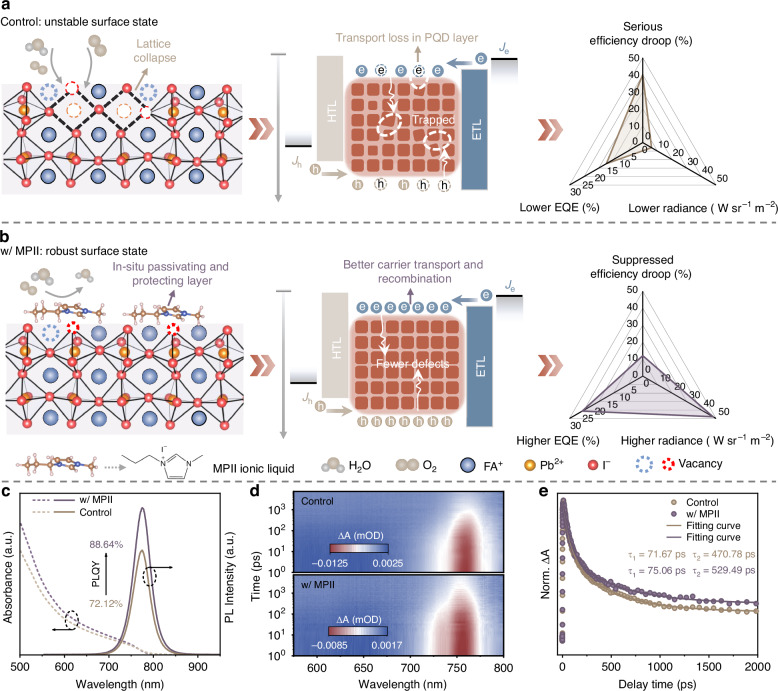
Ionic liquid-mediated surface reconstruction strategy for PQDs **a** Schematic diagram exhibiting the problems for pristine PQDs, as well as the carrier dynamics and device performance for the corresponding NIR LEDs. **b** Schematic diagram showing the proposed ionic liquid-mediated surface state reconstruction solution for PQDs to enable defect passivation and carrier transport regulation for achieving better device performance. **c** UV-Vis absorption and PL spectra of control and MPII-treated PQDs. **d**, **e** The fs-TA spectra, and normalized decay kinetics of the GSB signal for control and MPII-treated PQDs

We synthesized FAPbI_3_ PQDs *via* a hot-injection method, following our previously established protocol^28,29^. During synthesis, long-chain ligands such as oleic acid (OA) and oleylamine (OAm) play essential roles in maintaining colloidal stability, halting PQD growth, and suppressing undesired ripening. However, during purification, ligand loss exposes the high-energy PQD surface, leading to severe aggregation and lattice collapse, which significantly degrades the optical properties and stability of PQDs^30^. As a result, the NIR LEDs fabricated using these untreated PQDs (hereafter control PQDs) suffer from numerous carrier quenching pathways and poor device performance (Fig. 1a). To address this, we introduced the ionic liquid MPII into the antisolvent during purification, producing MPII-treated PQDs (w/ MPII PQDs). Due to its intrinsic liquid nature, MPII is highly soluble in nonpolar antisolvents, minimizing the surface damage typically caused by polar solvents and thus enabling the preservation of a stable PQD surface state (Fig. 1b).

We first employed transmission electron microscopy (TEM) to investigate the morphological effects of MPII treatment on PQDs. Compared with the control PQDs, the MPII-treated PQDs retain their cubic shape and uniform dispersion (Fig. S1). This improvement is mainly attributed to the surface reconstruction facilitated by MPII during purification, which effectively suppresses PQD aggregation and lattice collapse^31^. Additionally, atomic force microscopy (AFM) measurements reveal that MPII-treated PQD films exhibit reduced root-mean-square (RMS) surface roughness (Fig. S2). The smoother film morphology is anticipated to mitigate localized carrier over-injection in PQD-based LEDs, thereby reducing efficiency roll-off and enhancing device performance^32^.

We next assessed the optical properties of PQDs through steady-state photoluminescence (PL) measurements. As illustrated in Fig. 1c, the MPII-treated PQDs exhibit a marked enhancement in PL intensity, with their PLQY increasing from 72.12% to 88.64%, indicating suppressed exciton quenching^28,30^. Time-resolved photoluminescence (TRPL) measurements of the PQD solution further revealed an increase in the average PL lifetime from 66.62 to 96.22 ns following MPII treatment, which can be attributed to the effective defect passivation that reduces the non-radiative recombination losses (Fig. S3a and Table S1). This result is consistent with the improved PLQY^33^. Notably, despite the average PL lifetime of the spin-coated control and MPII-treated PQD films decreasing compared with their corresponding solution, the reduction in average lifetime for the MPII-treated films was much less pronounced than that of the control films (Fig. S3b and Table S2). This result indicates that the strong ligand-surface interaction of MPII effectively preserves the integrity of the surface ligands, thereby mitigating the formation of new defects during the spin-coating process.

We then conducted the femtosecond transient absorption (fs-TA) measurements to further investigate the carrier recombination dynamics of PQDs. We extracted the decay kinetics from the ground-state bleaching (GSB) signal centered at ~758 nm (Fig. 1d and S4), which corresponds to the band edge absorption of FAPbI_3_ PQDs. The biexponential fitting of the kinetic traces revealed a short time constant *τ*_1_ of 75.06 ps and a long time constant τ_2_ of 529.49 ps for MPII-treated PQDs. In contrast, faster decay kinetic processes (*τ*_1_ = 71.67 ps, *τ*_2_ = 470.78 ps) were observed for the control PQDs (Fig. 1e and Table S3). Typically, the hot-exciton cooling process occurs within several hundred femtoseconds to a few picoseconds. Therefore, the fitted short (*τ*_1_) and long lifetimes (*τ*_2_) can be attributed to exciton trapping and band edge exciton recombination processes, respectively^28^. The prolonged lifetimes observed in MPII-treated PQDs indicate a lower trap density and more efficient exciton utilization compared to the control PQDs, consistent with the improved defect passivation in other optical measurements.

**Fig. 2:**
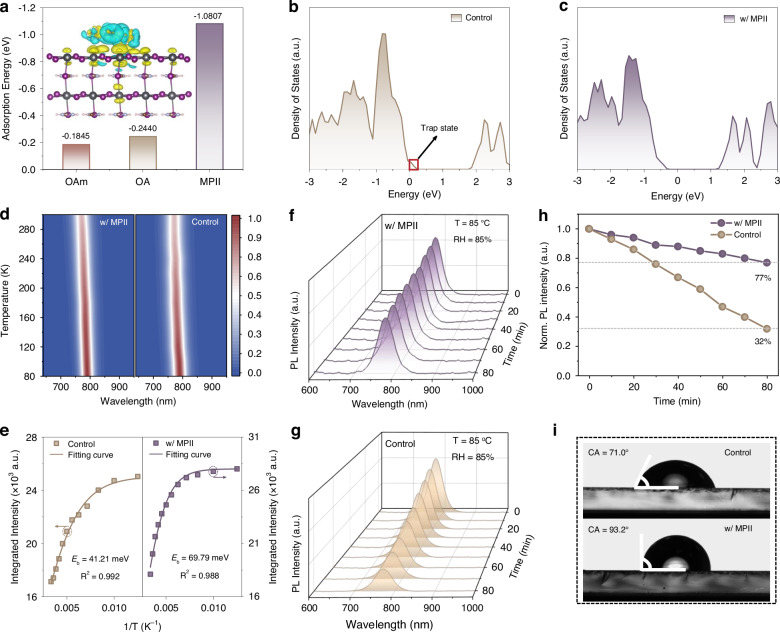
Trap state and optical characteristics of PQDs **a** The calculated binding energy of various ligands to the PQD surface, the inset exhibits the differential charge density mapping of MPII-treated PQDs. **b**, **c** The density of states (DOS) of control and MPII-treated PQDs. **d** Temperature-dependent PL spectra, and **e** the corresponding integrated PL intensity as a function of temperature for control and MPII-treated PQD films. **f**, **g** PL spectra as a function of aging time for control and MPII-treated PQD films under the conditions of 85 °C and relative humidity (RH) of 85%. **h** The evolution of PL intensity for control and MPII-treated PQD films. **i** Contact angle (CA) of the control and MPII-treated PQD films

To further understand the effect of MPII treatment on defect passivation, we employed density functional theory (DFT) simulation to calculate the binding affinity of MPII and oleate ligands (OA, OAm) on the PQD surface. The results show that the binding energy of MPII (-1.0807 eV) is nearly 5-fold higher than that of oleate ligands, indicating a much stronger ligand-surface interaction (Fig. 2a and S5). Additionally, the differential charge density mapping (inset of Fig. 2a) reveals clear evidence of electron transfer, demonstrating a strong interaction between the electron-rich imidazole ring of MPI^+^ cations and uncoordinated Pb^2+^ions^34,35^. We also investigated the energy band structure of PQDs by simulating the density of states (DOS). As shown in Fig. 2b, the PQDs with FA^+^ and I^-^ vacancies (V_FA_, V_I_) introduce shallow-level trap states near the valence band maximum, which may hinder carrier transport and promote non-radiative recombination. By contrast, for MPII-treated PQDs, these shallow-level trap states were thoroughly diminished (Fig. 2c)^31,36^.

X-ray photoelectron spectroscopy (XPS) measurements were performed to elucidate the interaction between MPII and PQDs. We adopted the C 1 s peak at 284.8 eV as the internal reference to calibrate all the obtained spectra. Compared to the control, the Pb 4 f and I 3 d peaks in MPII-treated PQDs shifted to lower binding energies, indicating altered surface chemical environments due to increased electron density from the electron-rich imidazole ring of MPI⁺ cations (Fig. S6)^34,35^. Fourier transform infrared (FTIR) spectroscopy revealed a C=N stretching vibration at 1562 cm^-1^ in MPII-treated PQDs, shifted from 1566 cm^-1^ in pure MPII, confirming MPI^+^ cations anchoring on the PQD surface (Fig. S7)^37^. Furthermore, the decreased intensities of C-H_*x*_ (*v* = 2760–3000 cm^-1^) and C=N-H_2_^+^ (*v* = 3210–3460 cm^-1^) vibrations suggest the partial displacement of long-chain oleate ligands upon MPII treatment^28^. To quantitatively evaluate the surface density of long-chain oleate ligands, we further conducted the ^1^H NMR measurements for pristine, control and MPII-treated PQDs. By calculating the integral value of the peak located at around 5.3 ppm, which corresponds to the chemical shift signals of octadecene and oleyl species, we found that the MPII treatment reduced the long-chain ligand density by ~85.8% (Fig. S8)^28^.

We next determined the exciton binding energy of PQDs by measuring temperature-dependent PL spectra (Fig. 2d). As the temperature decreased from 300 to 80 K, we observed a significant enhancement in the integrated PL intensity for both PQD films (Fig. S9), which can be attributed to the suppression of exciton dissociation and non-radiative recombination capture at lower temperatures. Subsequently, we plotted the integrated PL intensity against temperature and extracted the exciton binding energy (*E*_b_) for each PQD film by fitting the data using the following equation^38^:

1 $$I\left(T\right)=\frac{{I}_{0}}{1\,+A{e}^{-\frac{{E}_{b}}{{k}_{B}T}}}$$

where, *I*_0_ is the integrated PL intensity at 80 K, *E*_b_ is the exciton binding energy, *k*_B_ is the Boltzmann constant, and *T* is the temperature. The *E*_b_ of MPII-treated PQD films was calculated as 69.79 meV, which is obviously higher than that of control PQD films (Fig. 2e). The higher exciton binding energy suggests that excitons are less likely to dissociate into free carriers, thereby increasing radiative recombination probability and contributing to device efficiency improvement^39^.

We further used in situ PL measurements to evaluate the PL stability of PQD films. Under accelerated aging conditions of 85 °C and a relative humidity (RH) of 85%, we recorded the PL spectra as a function of time. In contrast to the rapid decrease in PL intensity of control PQD films, we found that MPII-treated PQD films maintained more than 70% of their initial PL intensity even after 80 min of aging (Fig. 2f–h, and S10). The improved PL stability can be attributed to the dual function of MPI⁺ cations: while passivating surface defects, their alkyl side chain simultaneously forms a hydrophobic protective layer on PQD surface, as supported by the increased water contact angle (Fig. 2i)^40^.

To gain an in-depth understanding of the improved water-oxygen and thermal stability of PQDs, we employed X-ray diffraction (XRD) measurements to track the crystalline phase evolution of aged PQD films (Fig. S11). We found that a diffraction peak at ~11°—assigned to the (010) crystal plane of non-photoactive δ-phase FAPbI_3_—emerged in control PQD films after 30 min of aging. In contrast, the δ-phase peak appeared only after 60 min in MPII-treated PQD films, and the relative diffraction peak intensity at ~11° remained significantly lower than that of control PQD films after 80 min of aging (Fig. S11), indicating that MPII treatment ensures better PQD phase stability^24,41^.

**Fig. 3:**
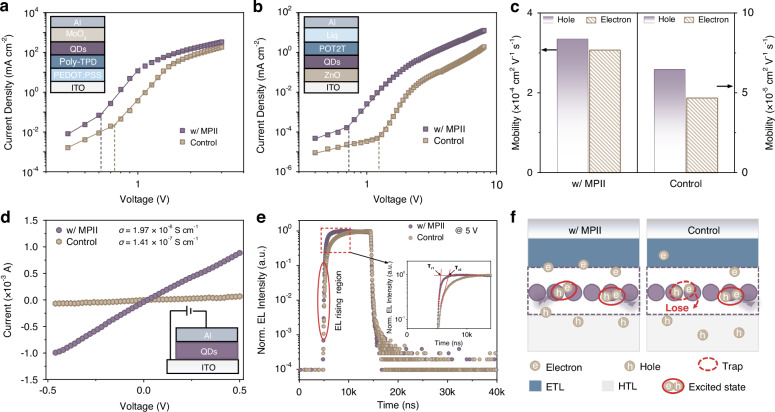
Carrier mobility analysis of PQD films **a**, **b** Space-charge limited current (SCLC) measurements of hole-only and electron-only devices. **c** Hole and electron mobility of control and MPII-treated PQD films. **d** Current-voltage (I–V) curves of control and MPII-treated PQD films. **e** Transient electroluminescence (TREL) decay curves of control and MPII-treated NIR LEDs. **f** Schematic diagrams of the corresponding electronic process indicated in Fig. 3e

Inspired by the improved optical properties and film stability, we next operated space-charge limited current (SCLC) measurements to further analyze the carrier mobility and trap density of PQD films under an electrical field. As illustrated in Fig. 3a, b, the hole and electron mobilities (*μ*) of PQD films were calculated from the child region of current density-voltage (J–V) curves using the following Mott-Gurney equation^42^:

2 $$\mu =\frac{8J{d}^{3}}{9{\varepsilon }_{r}{\varepsilon }_{0}{V}^{2}}$$

where, *J*, *d*, and *V* represent the dark current density, thickness of PQD films, and applied voltage, respectively. ${\varepsilon }_{0}$ and ${\varepsilon }_{r}$ are vacuum permittivity (${\varepsilon }_{0}$ = 8.854 × 10^-14 ^F cm^-1^) and relative permittivity of perovskite (46.9 for FAPbI_3_)^28,29^. We obtained a hole mobility of 3.35 × 10^-4 ^cm^2 ^V^-1^ s^-1^ and electron mobility of 3.07 × 10^-4 ^cm^2 ^V^-1^ s^-1^ for MPII-treated PQD films, which is nearly 5.2-fold and 6.5-fold higher than those of the control films (6.46 × 10^-5 ^cm^2 ^V^-1^ s^-1^ and 4.67 × 10^-5 ^cm^2 ^V^-1^ s^-1^), respectively (Fig. 3c). Furthermore, the small difference between hole and electron mobilities in MPII-treated PQD films confirms more balanced carrier injection, which is essential for improving device performance.

According to the trap-filled limit voltage extracted from the J-V curves of electron-only devices, we also determined the trap density (*n*_*trap*_) of PQD films using the following equation^42^:

3 $${n}_{{trap}}=\frac{2{\varepsilon }_{r}{\varepsilon }_{0}{V}_{{TFL}}}{e{d}^{2}}$$

where, *V*_*TFL*_ is the trap-filled limit voltage, *e* stands for the elemental charge, and *d* is the thickness of QD films. The estimated trap density of MPII-treated PQD films is 3.73 × 10^17 ^cm^-3^, nearly 1.7-fold lower than that of control films (6.38 × 10^17 ^cm^-3^), confirming effective defect passivation by MPII.

We also evaluated the conductivity of PQD films by building a two-terminal device with an indium tin oxide (ITO)/PQDs/Al architecture (Fig. 3d). The resulting current-voltage (I–V) curves for both PQD films exhibit a linear characteristic in the scanning voltage range. We calculated the conductivity (*σ*) for each film according to the following equation^43,44^:

4 $$\sigma =\frac{{Id}}{{AV}}$$

where, *I* is the current intensity, *V* is the applied voltage, *d* is the thickness of PQD films, and *A* is the device area. The calculated *σ* for MPII-treated PQD films is 1.97 × 10^-6^ S cm^-1^, more than 10-fold higher than that of control films (1.41 × 10^-7^ S cm^-1^). In addition, we further conducted the transient electroluminescence (TREL) measurements to examine the response speed of the fabricated PQD-based NIR LEDs under a pulse voltage of 5 V (Fig. 3e). We observed that MPII-treated devices exhibit a faster response to the electrical pulse compared with the control devices, as evidenced by the more rapid increase in EL intensity (τ_r1_ < τ_r2_) in the EL rising region, indicating superior charge transfer capability. This can be attributed to the reduced trap states in the MPII-treated PQDs, which minimizes defect-mediated carrier trapping during charge injection, as schematically illustrated in Fig. 3f^45^.

**Fig. 4:**
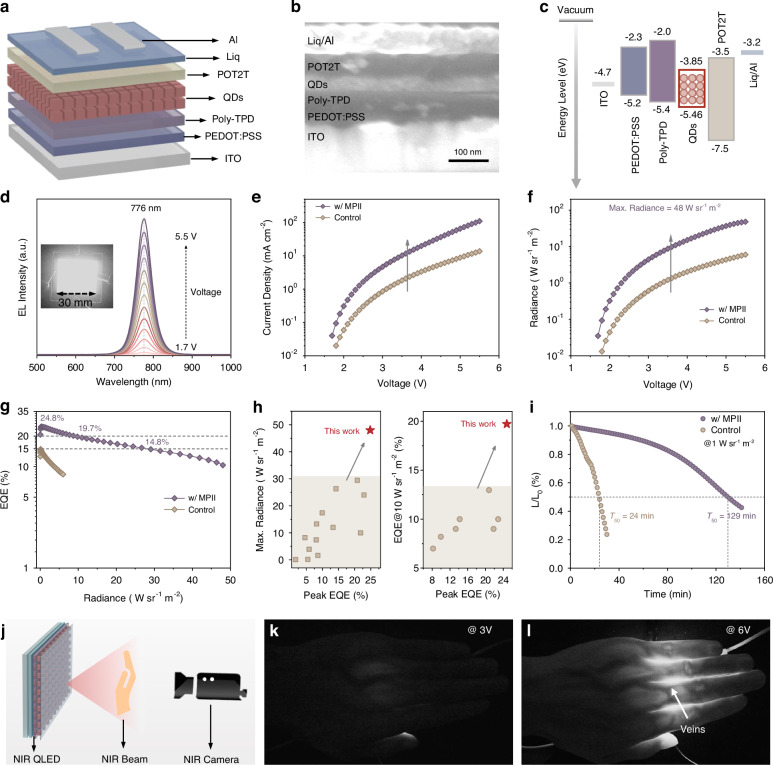
Device performance of MPII-treated PQD-based NIR LEDs **a**–**c** Device structure, cross-sectional scanning electron microscopy (SEM) image, and energy level diagram of the fabricated PQD-based NIR LEDs. **d** Electroluminescent (EL) spectra of MPII-treated PQD-based NIR LEDs, the inset shows a picture of lighted NIR LEDs with an active area of 900 mm^2^. **e** Current density-voltage curves, **f** radiance-voltage curves, and **g** EQE-radiance curves of the control and MPII-treated PQD-based NIR LEDs. **h** Summary of reported PQD-based NIR LEDs characteristics on the basis of peak EQE and maximum radiance, or EQE@10 W sr^-1^ m^-2^ (the corresponding data are listed in Table S4). **i** Operational stability of the control and MPII-treated PQD-based NIR LEDs tested in the ambient environment. **j** Schematic diagram of palm veins imaging with MPII-treated PQD-based LEDs as NIR light source. **k**, **l** Photographs of the palm taken under the irradiation of MPII-treated PQD-based NIR LEDs with different driving voltages

Encouraged by the significantly improved optical and electrical properties, we employed MPII-treated PQD films as the emitting layer to fabricate the PQD-based NIR LEDs. We adopted a device structure with ITO glass substrate as the anode, poly(3, 4-ethylene-dioxythiophene):polystyrene sulfonate (PEDOT:PSS) as the hole injection layer, poly[N, N’-bis(4-butylphenyl)-N, N’-bis(phenyl)-benzidine] (Poly-TPD) as the hole transport layer, 2, 4, 6-tris[3-(diphenylphosphine) phenyl]-1, 3, 5-triazine (POT2T) as the electron transport layer, and Liq/Al as the double layered cathode (Fig. 4a). The device cross-sectional SEM image exhibited in Fig. 4b clearly shows each layer. Additionally, the energy level diagram of the device was determined with the aid of UPS measurement results (Fig. 4c and S12).

The resulting PQD-based NIR LEDs exhibit stable electroluminescence (EL) centered at 776 nm, with no spectral shift observed over a voltage range of 1.7-5.5 V (Fig. 4d). MPII-treated devices demonstrate enhanced conductivity and significantly higher current density, which is consistent with the improved carrier transport in MPII-treated PQD films (Fig. 4e). Furthermore, these PQD-based NIR LEDs achieve a maximum radiance of 48 W sr^-1^ m^-2^, nearly 8-fold higher than that of the control devices (Fig. 4f). Notably, MPII-treated devices reach a record peak EQE of 24.8% with markedly suppressed efficiency roll-off: EQE remains ~20% at 10 W sr^-1^ m^-2^ and ~15% at 30 W sr^-1^ m^-2^ radiance (Fig. 4g). By contrast, control devices exhibit a lower peak EQE of 14.9% and suffer a ~ 40% EQE drop at 5 W sr^-1^ m^-2^ (Fig. 4g). The performance of MPII-treated devices ranks among the highest reported for PQD-based NIR LEDs to data (Fig. 4h and Table S4). To verify reproducibility, thirty MPII-treated PQD-based NIR LEDs were fabricated, showing an average EQE of 22.45% and radiance of 45.57 W sr^-1^ m^-2^ with low deviation (Fig. S13). Operational stability tests yielded a half-lifetime (*T*_50_) of 129 min for MPII-treated devices at an initial radiance of 1 W sr^-1^ m^-2^, 5-fold longer than control devices (Fig. 4i).

Building on our strategy for high-quality PQDs, we fabricated large-area PQD-based NIR LEDs (active area: 900 mm^2^) *via* blade-coating. These devices exhibit uniform NIR emission with a peak EQE of 19.9% and maximum radiance of 9.5 W sr^-1^ m^-2^, demonstrating promising application potential (inset of Fig. 4d and S14). We further demonstrated noninvasive bioimaging using these NIR LEDs (Fig. 4j). Leveraging the NIR light’s tissue penetration and hemoglobin absorption properties, we successfully imaged human palm veins (Fig. 4k, l)^46^. Notably, image resolution improved substantially when increasing the driving voltage from 3 V (EQE ~ 19%, radiance 1 W sr^-^¹ m^-^²) to 6 V (EQE ~ 18%, radiance 9.5 W sr^-^¹ m^-^²), highlighting the importance of both high EQE and radiance for practical applications.

**Fig. 5:**
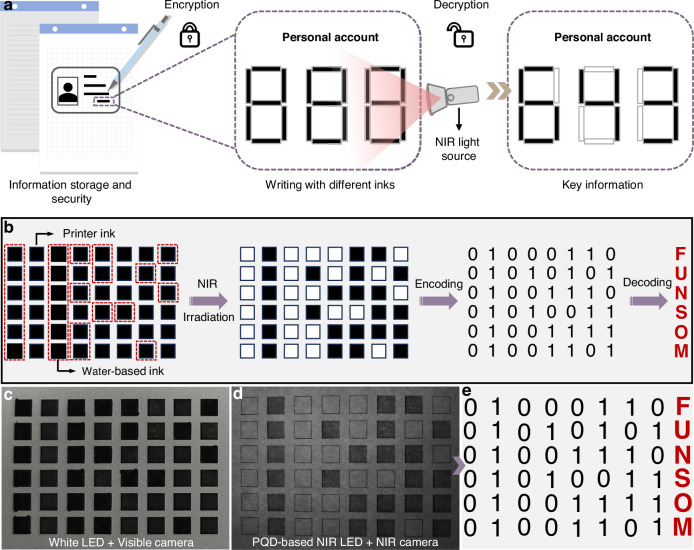
Frontier application of MPII-treated PQD-based NIR LEDs **a** Schematic illustration of the application principle of NIR light sources in information storage and security. **b** Schematic diagram of the dot patterns printed with different inks for achieving information encryption and decryption with the assistance of MPII-treated PQD-based NIR LEDs. **c**, **d** Images of the dot patterns captured under the illumination of white LEDs and MPII-treated PQD-based NIR LEDs, respectively. **e** The obtained binary-coded information from Fig. 5d

We also explored their use as NIR light sources for information encryption and decryption, exploiting the differing NIR absorption characteristics of printer and water-based inks. The invisibility of the water-based ink under NIR illumination originates from its negligible absorption in the NIR region, whereas printer inks containing carbon-based pigments exhibit strong NIR absorption. Therefore, the patterns printed with water-based ink become invisible under NIR illumination, whereas those printed with printer ink remain detectable via an NIR camera^47^. Using this principle, we encoded hidden binary information in a 6 × 8 black square dot matrix printed with both inks (Fig. 5a, b). Under white LED illumination, all dots were visible in a visible-light camera image (Fig. 5c). Under irradiation with the large-area PQD-based NIR LEDs, only the printer ink dots were detected by an NIR camera, yielding a binary black-and-white matrix (Fig. 5d). Interpreting black and white dots as “1” and “0” respectively, we successfully decoded the concealed message as “FUNSOM” (Fig. 5e).

## Discussion

In summary, we present an ionic liquid-mediated surface reconstruction strategy for PQDs. By introducing a multifunctional ionic liquid MPII in the antisolvent, defects are effectively passivated, and a protective layer is formed in situ, yielding PQDs with stabilized surface states. This approach enables PQD films with reduced trap density, enhanced conductivity, and improved phase stability. Leveraging these advances, we fabricated the PQD-based NIR LED,s achieving a record peak EQE of 24.8% with minimal efficiency roll-off, maintaining ~20% EQE at a radiance of 10 W sr^-1^ m^-2^—the highest reported for PQD-based NIR LEDs. Moreover, efficient large-area devices (900 mm²) with an EQE up to 20% were demonstrated, alongside practical applications in bioimaging and information encryption. Our strategy paves the way for advancing high-performance PQD-based NIR LEDs and their emerging applications.

## Materials and methods

### Materials

The raw materials utilized in synthesis including formamidine acetate (HN=CHNH_2_ ∙ CH_3_COOH, 99%, Sigma-Aldrich), lead (II) iodide (PbI_2_, 99.999%, Adamas), 1-octadecene (ODE, 90%, Adamas), oleic acid (OA, 90%, Adamas), oleylamine (OAm, 90%, Adamas), octane (anhydrous, 99%, Adamas), methyl acetate (MeOAc, ≥98.0%, Greagent), 1-methyl-3-propylimidazolium iodide (MPII, Sigma-Aldrich). The PEDOT:PSS (AI4083), Poly-TPD used to fabricate PQD-based NIR LEDs were purchased from Xi’an Yuri Solar Co., Ltd. China. POT2T was purchased from Luminescence Technology Corp. The indium tin oxide (ITO) glass substrates were provided by South China Xiangcheng Technology Co., Ltd. China. The ITO glass substrate was patterned to generate four devices, and each with an active area of 10 mm^2^. All the materials were used as received without further purification.

### Synthesis of FAPbI_3_ PQDs

FAPbI_3_ PQDs were prepared using a hot injection method. In a typical synthesis, 6 mmol FA(Ac) was weighed and transferred to a 50 mL three-necked flask containing 12 mL OA. The reaction mixture was stirred and degassed for 5 min at room temperature, then the reaction system was equably heated to 110 °C and continued to be degassed for 40 min until FA(Ac) was completely dissolved. In another flask containing a mixture of 2 mL OA, 2 mL OAm and 20 mL ODE, 0.8 mmol PbI_2_ was added and stirred at room temperature for 5 min to degas. After that, the reaction system was heated to 100 °C and continued to be degassed for 40 min until the clear lead halide precursor was formed. Before hot injection, the temperature of the FA-oleate precursor and lead halide precursor was reduced to 100 °C and 80 °C under a nitrogen (N_2_) atmosphere, respectively. Then, 4.0 mL FA-oleate precursor solution was rapidly injected into the flask containing lead halide precursor, and the solution was then quickly cooled down to room temperature with an ice-water bath. After the reaction, the solution was centrifuged at 7800 rpm for 10 min to eliminate the unreacted precursors. Subsequently, the precipitate was collected and dissolved in octane to obtain a FAPbI_3_ PQD crude solution.

### Purification of FAPbI_3_ PQDs

First, 2.5 mL MeOAc was added to the FAPbI_3_ PQD crude solution. Then, the resulting mixture was centrifuged for 5 min to collect the supernatant. Subsequently, an additional 2.5 mL MeOAc was added to the collected supernatant, followed by a second centrifugation. Finally, the precipitate obtained after this step was collected and dissolved in octane to yield the purified PQD solution. The prepared PQD solution was stored at 4 °C until further use.

### MPII ionic liquid-mediated surface reconstruction strategy

During the purification process, the second added pure MeOAc was replaced with 0.2 mg mL^-1^ MPII solution (prepared by mixing the MPII ionic liquid with MeOAc). After the centrifugation, the precipitate was collected and dissolved in octane to obtain the MPII-treated FAPbI_3_ PQDs, and the treated PQD solution was also stored at 4 °C for further use.

### Fabrication of PQD-based NIR LEDs

The patterned ITO glass substrates were first cleaned using ethanol and deionized water, respectively. Before fabricating the devices, the ITO glass substrates need to be dried with nitrogen (N_2_) stream and treated with UV-ozone for 15 min. After that, PEDOT:PSS was spin-coated onto the ITO glass substrate at 4000 rpm for 40 s, followed by annealing on a heating stage at 150 °C for 20 min. After annealing, when the temperature of the ITO glass substrate was cooled to room temperature, the Poly-TPD chlorobenzene solution (8 mg mL^-1^) was spin-coated onto the PEDOT:PSS layer at 4000 rpm for 40 s, and followed by annealing at 100 °C for 15 min in the glove box. Subsequently, the FAPbI_3_ PQD solution (20 mg mL^-1^) was spin-coated onto the Poly-TPD layer at 3000 rpm for 30 s. Finally, the POT2T (∼60 nm), Liq (2 nm), and Al (∼80 nm) layers were deposited in turn using a thermal evaporation system (Suzhou Fangsheng FS380-S12) under a high vacuum (<10^-4 ^Pa). As for the large-area PQD-based NIR LEDs, the hole transport and PQD layers were fabricated by blade-coating. The PEDOT:PSS, Poly-TPD, and PQD layers were spread utilizing a knife with a gap of 200 μm at a speed of 20 mm s^-1^. Then, POT2T (∼60 nm), Liq (2 nm), and Al (∼80 nm) electrodes were thermally evaporated on the top of the devices under a vacuum below 10^-4 ^Pa.

### Characterization

The UV–Vis absorption spectra were tested using a Lambda 750 UV-VIS-NIR spectrophotometer. The photoluminescence (PL) spectra were measured utilizing a FL3 NIR-VIS fluorescence spectrometer. The temperature-dependent PL spectra were recorded using the above FL3 NIR-VIS fluorescence spectrometer equipped with a constant temperature heating instrument. A fiber spectrophotometer system (AVANTES Avaspec Mini 2048CL-SHB3) equipped with a 460 nm laser as excitation source was used to measure the PL spectra of PQD films at different times under the aging conditions of 85 °C and 85% RH. The photoluminescence quantum yields (PLQYs) of PQDs were obtained using an integration sphere system equipped with a 405 nm laser as excitation source and a spectrometer (QE Pro-FL, Ocean Optics) with a detection range from 350 to 1100 nm. Time-resolved photoluminescence (TRPL) decay curves were recorded by a HORIB-FM-2015 fluorescence spectrometer. The femtosecond transient absorption (fs-TA) measurements were performed by a Helios pump-probe system (Ultrafast Systems) integrated with a regenerative amplified laser source (Coherent). The Ti: sapphire laser amplifier (Astrella, Coherent). The X-ray photoelectron spectroscopy (XPS) and ultraviolet photoelectron spectroscopy (UPS) measurements were performed using a KRATOS AXIS Ultra DLD with a base pressure of ~10^-9^ torr. Fourier transform infrared spectroscopy (FTIR) spectra were measured using a VERTEX 70 FT-IR spectrometer under transmission mode. The ^1^H NMR spectra were tested using a Bruker nuclear magnetic resonance spectrometer with a frequency of 400 MHz. The transmission electron microscopy (TEM) images were captured using a Talos F200X electron microscope. The atomic force microscopy (AFM) images were acquired using a Cypher-S atomic force microscope (Asylum Research, Oxford Instruments, UK). The contact angle of PQD films was tested utilizing a Data-Physics OCA system. The accelerated aging test was implemented in a constant temperature and humidity chamber (BPS-50CL, Blue pard), maintaining an atmosphere of high temperature (85 °C) and high humidity (85%). The X-ray diffraction (XRD) measurements were conducted using a Bruker AXS D8 diffractometer equipped with Cu Kα radiation (*λ* = 1.54178 Å). The test parameters, including tube voltage, current, angular range, and scanning rate, were set to 40 kV, 40 mA, 5°–50°, and 10° min^-1^, respectively.

All the PQD-based NIR LEDs were characterized under ambient conditions (*T* = 24–28 °C, RH = 40–50%). The transient electroluminescence (TREL) decay curves were measured under an electrical excitation with a pulse width of 4 µs generated by a pulse generator (Keysight 81150 A). The current density-voltage (J–V) characteristics of different types of devices were recorded using a computer-controlled Keithley 2400 source meter. The external quantum efficiency (EQE) and electroluminescence (EL) spectra were tested using a calibrated photonic multi-channel PMA-12 analyzer system (Hamamatsu C10027-01 (360–950 nm)). The PMA-12 system was connected to an integrating sphere (3.3 in, collecting device forward light) and a power supply system (controlling current output), while a PR-745 instrument (Photo Research, 380-1060 nm) was adopted to calibrate the absolute radiance. All the measurements assumed Lambertian emission. The photographs of the lighted large-area devices and the application demonstration images were taken using a camera with natural and NIR modes (XA10E, Canon, Japan).

### Density functional theory calculation

Density functional theory (DFT) as implemented in the Vienna ab initio simulation package (VASP), was used to conducted the calculation presented in this work. The projector augmented wave (PAW) method was utilized to treat the effective interaction of the core electrons and nucleus with the valence electrons, while exchange and correlation were described using the Perdew-Burke-Ernzerhof (PBE) functional. The cut-off energy is set at 400 eV for the plane-wave basis restriction in all calculation. K-points are sampled under the Monkhorst-Pack scheme for the Brillouin-zone integration (K-points were sampled using the Gamma Point). A K-point mesh of 1 × 1 × 1 was adopted for the structure relaxation of all slab models, while a K-point mesh of 9 × 1 × 9 was adopted for density of states (DOS) calculations. In all calculations, the forces acting on all atoms are <0.02 eV Å^-1^ in fully relaxed structures, and self-consistency accuracy of 10^-5 ^eV is reached for electronic loops. The binding energy (*E*_binding_) was calculated following the equation *E*_binding_ = *E* – *E*_a_ – *E*_b_, where *E* is the total energy of the adsorbed system, *E*_a_ and *E*_b_ stand for the total energy of tree species and the bare surface, respectively.

## Supplementary information

Supplemental Information

## Data Availability

The data that support the plots within this paper and other findings of this study are available from the corresponding author upon reasonable request.
