# Supplementary material for: Ion agent mitigates efficiency roll-off in near-infrared electroluminescence for practical bioimaging and information encryption
Source: Light Sci Appl. 2026 May 7;15:221. doi: 10.1038/s41377-026-02237-1 (PMC13153217; doi:10.1038/s41377-026-02237-1)
Supplement: Supplementary file 1 — Supplemental Information [file 41377_2026_2237_MOESM1_ESM.docx]

Supplementary Information for

**Ion agent mitigates efficiency roll-off in near-infrared electroluminescence for practical bioimaging and information encryption**

Tao Yang^1^, Ye Wang^1^, Zong-Shuo Liu^1^, Feng Zhao^1^, Wei-Zhi Liu^1^, Wan-Shan Shen^1^, Ya-Kun Wang^1^*, and Liang-Sheng Liao^1,2^*

*^1^Institute of Functional Nano & Soft Materials (FUNSOM), Jiangsu Key Laboratory for Carbon-Based Functional Materials & Devices, State Key Laboratory of Bioinspired Interfacial Materials Science, Soochow University, Suzhou 215123, Jiangsu, China.*

*^2^Macao Institute of Materials Science and Engineering, Macau University of Science and Technology, Macau SAR, Taipa 999078, China.*

* Email address: wangyakun@suda.edu.cn; lsliao@suda.edu.cn

**Contents**

1. **Supporting Tables**
2. **Supporting Figures**
3. **References**
4. **Supporting Tables**

**Table S1.** The detailed fitting parameters of TRPL decay curves for PQD solution.

| Sample | A_1_ | τ_1_ (ns) | A_2_ | τ_2_ (ns) | τ (ns) |
| --- | --- | --- | --- | --- | --- |
| Control | 2.91 | 28.54 | 0.71 | 100.10 | 66.62 |
| w/ MPII | 1.16 | 32.19 | 0.88 | 119.04 | 96.22 |

**Table S2.** The detailed fitting parameters of TRPL decay curves for PQD films.

| Sample | A_1_ | τ_1_ (ns) | A_2_ | τ_2_ (ns) | τ (ns) |
| --- | --- | --- | --- | --- | --- |
| Control | 5.73 | 19.24 | 0.46 | 80.28 | 34.56 |
| w/ MPII | 1.36 | 52.00 | 0.55 | 113.57 | 80.88 |

**Table S3.** The detailed fitting parameters of decay kinetics of GSB signal for PQDs.

| Sample | A_1_ | τ_1_ (ps) | A_2_ | τ_2_ (ps) | τ (ps) |
| --- | --- | --- | --- | --- | --- |
| Control | 0.43 | 71.67 | 0.42 | 470.78 | 416.96 |
| w/ MPII | 0.43 | 75.06 | 0.36 | 529.49 | 463.69 |

**Table S4.** Performance summary of previously reported NIR LEDs fabricated based on QDs.

| Materials | Emission Peak (nm) | EQE (%) | Max. Radiance (W sr^-1^ m^-2^) | EQE@10 W sr^-1^ m^-2^ (%) | Reference |
| --- | --- | --- | --- | --- | --- |
| In(Zn)As/In(Zn)P/  GaP/ZnS | 857 | 4.6 | 8.2 | **/** | [1] |
| InAs/ZnSe QDs | 947 | 5.5 | 0.15 | / | [2] |
| InAs/ZnSe QDs | 896 | 13.3 | 12 | 9^†^ | [3] |
| CuInS_2_/ZnS QDs | 940 | 8.2 | 13.3 | 7^†^ | [4] |
| PbS QDs in CsPbBr_3_ layer | 980 | 8.1 | 7.4 | / | [5] |
| Si NCs | 853 | 8.6 | 1.63 | / | [6] |
| Cs*_x_*FA*_1−x_*Pb(Br*_1−y_*I*_y_*)_3_ PQDs | 735 | 5.9 | 3.9 | / | [7] |
| FAPbI_3_:Sn^2+^ PQDs | 798 | 1.7 | 0.1 | / | [8] |
| FAPbI_3_:Mg^2+^ PQDs | 774 | 9.95 | 17.39 | 8.2^†^ | [9] |
| FAPbI_3_ PQDs | 742 | 14.2 | 26.3 | 10^†^ | [10] |
| FAPbI_3_ PQDs | 780 | 20.79 | 29.47 | 12.97^†^ | [11] |
| FAPbI_3_ PQDs in quasi-2D perovskite | 772 | 21.82 | 10 | 9^†^ | [12] |
| FAPbI_3_ PQDs | 776 | 22.86 | 24 | 10^†^ | [13] |
| FAPbI_3_ PQDs | 776 | 24.8 | 48 | 19.7 | This work |

^†^Estimated data from the figure in the references.

1. **Supporting Figures**

**
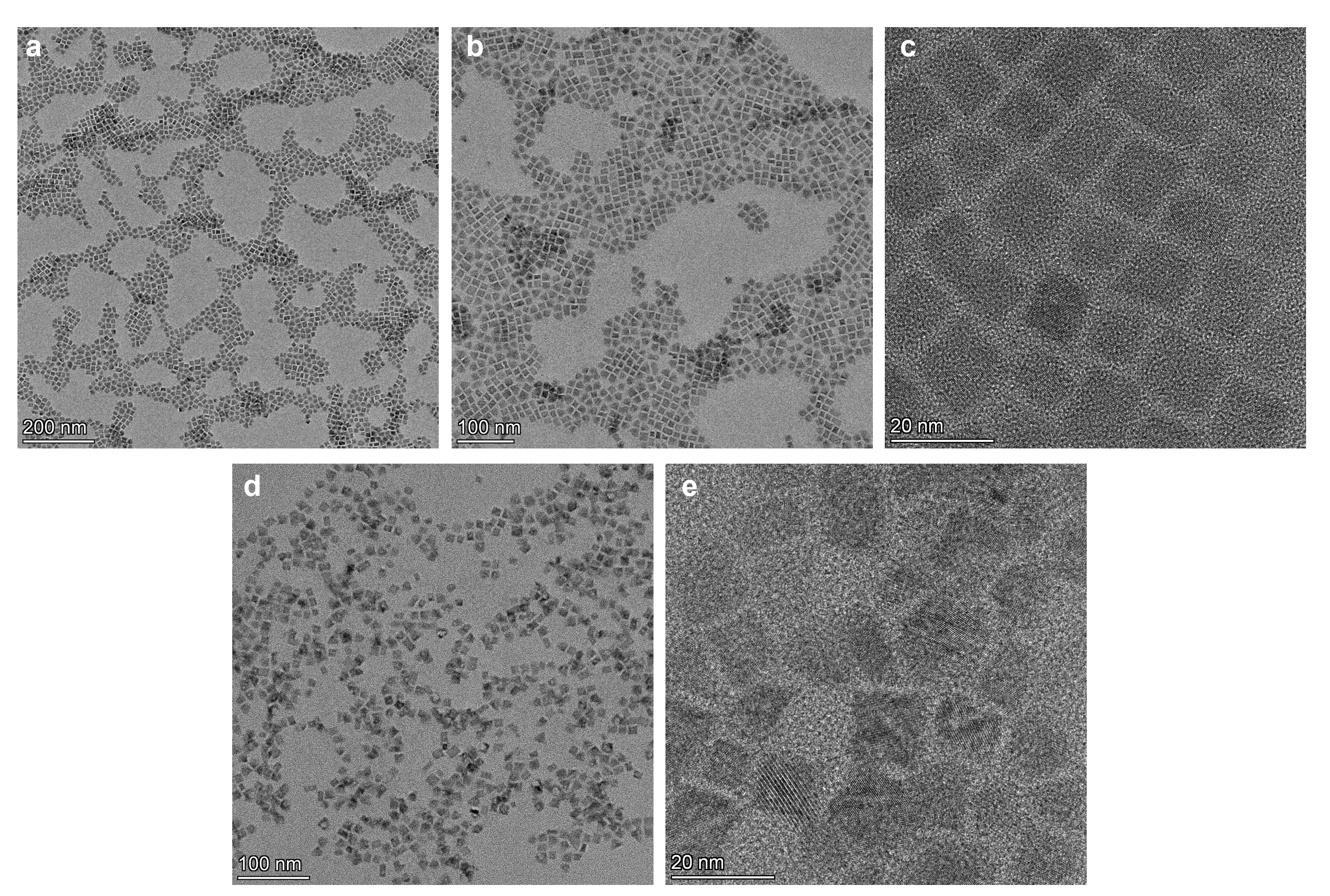
**

**Fig. S1** TEM images of (a-c) MPII-treated and (d, e) control PQDs.

**
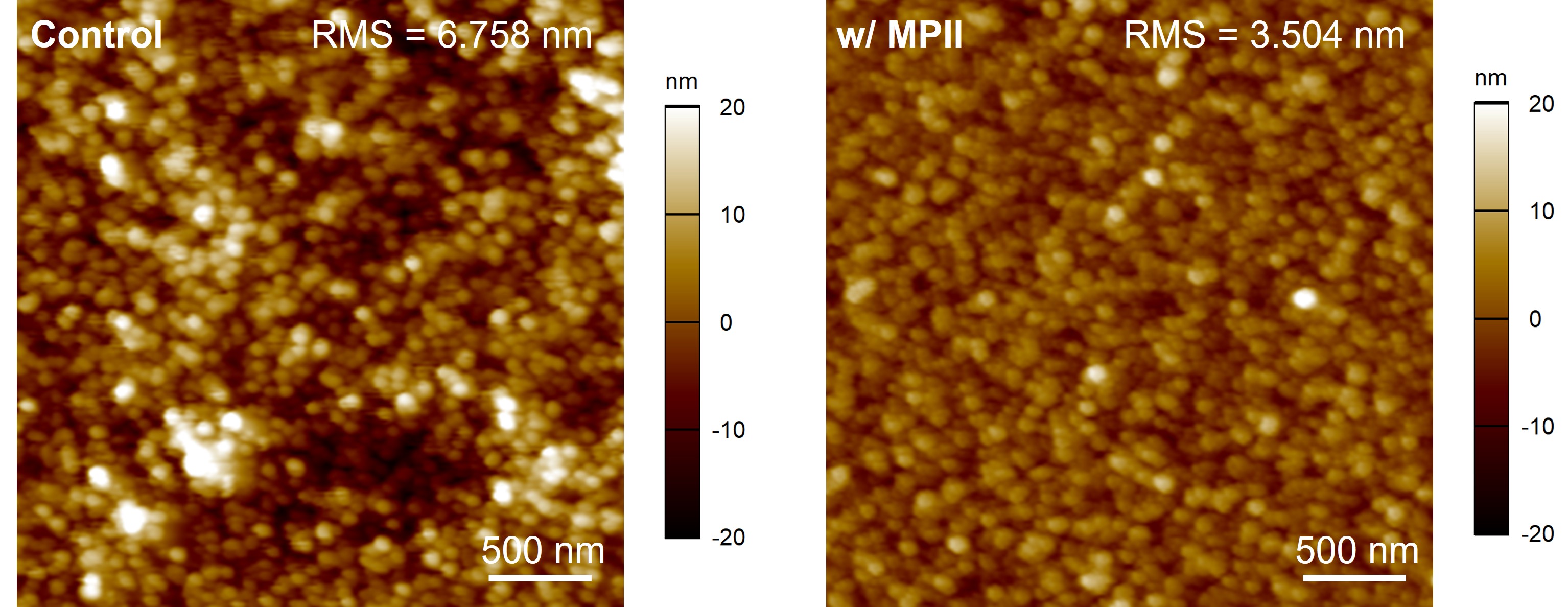
**

**Fig. S2** AFM images of the control and MPII-treated PQD films.

**

**

**Fig. S3** TRPL decay curves of the control and MPII-treated PQD (a) solution, and (b) films (the average lifetimes are fitted using the biexponential equation, the detailed fitting parameters are listed in Table S1 and S2).

**

**

**Fig. S4** The fs-TA spectra of (a) control, and (b) MPII-treated PQDs.

**
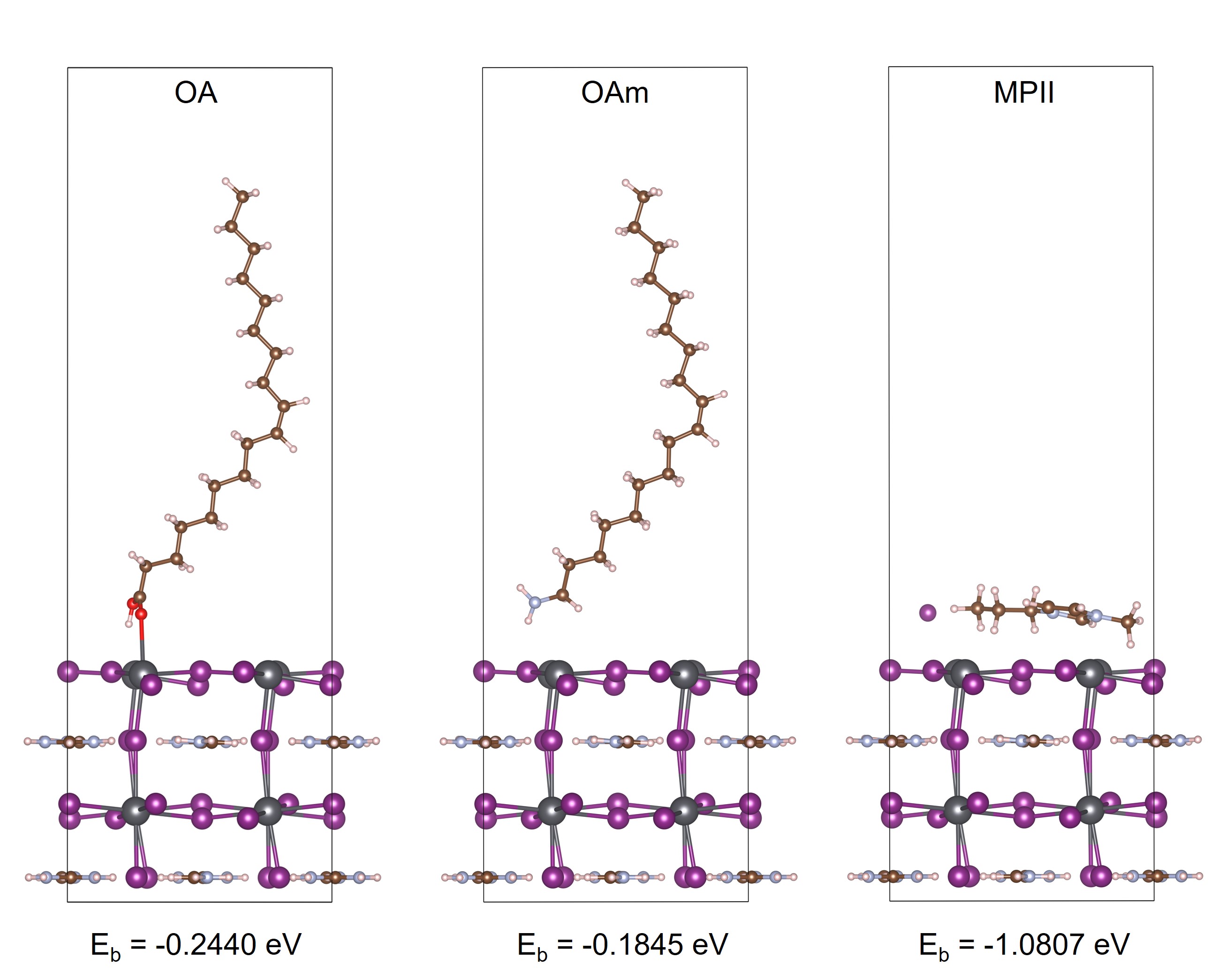
**

**Fig. S5** Theoretical calculations of the binding affinity of oleate ligands (OA, OAm) and MPII on the surface of FAPbI_3_ PQDs.





**Fig. S6** XPS characterization of PQDs before and after MPII treatment. (a) XPS survey spectra, (b) the calibrated C 1s spectra, (c) Pb 4f spectra, (d) I 3d spectra.





**Fig. S7** FTIR spectra of control, MPII-treated PQDs, and pure MPII.





**Fig. S8** (a) ^1^H NMR spectra of pristine, control and MPII-treated PQDs. (b) Local magnification from 0.6 to 0.8 ppm.





**Fig. S9** Temperature-dependent PL spectra of (a) MPII-treated, and (b) control PQD films measured at different temperatures (80-300 K).





**Fig. S10** PL spectra of the (a) MPII-treated, and (b) control PQD films measured at different times under the aging conditions of 85 ^o^C and a relative humidity (RH) of 85%.

**
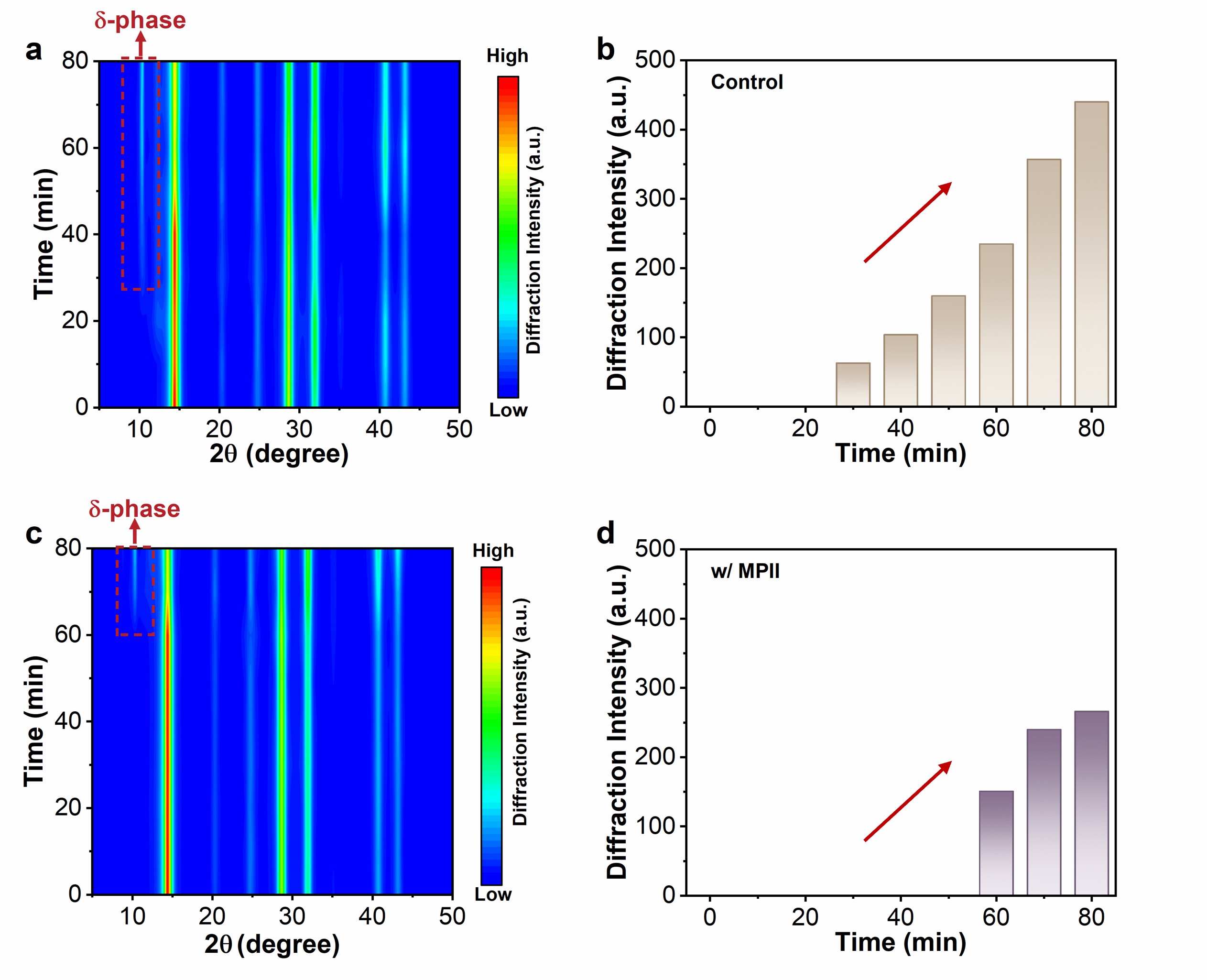
**

**Fig. S11** Evolution of the XRD patterns of (a) control, and (c) MPII-treated PQD films. Figures (b) and (d) exhibit the diffraction peak intensity of (010) crystal plane extracted from the corresponding XRD patterns.

**

**

**Fig. S12** (a) The Tauc plot extracted from the corresponding absorption spectra, and (b) UPS spectra of MPII-treated PQD films.





**Fig. S13** (a) Peak EQE, and (b) radiance histograms of thirty MPII-treated PQD-based NIR LEDs.

**
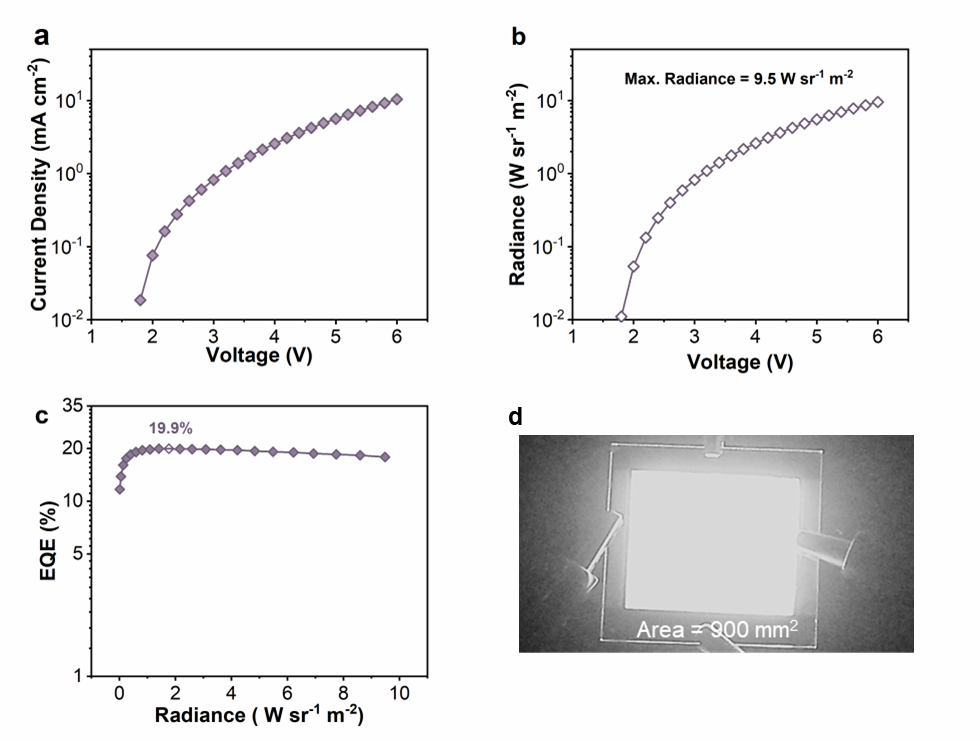
**

**Fig. S14** Device performance of the large-area NIR LEDs fabricated based on MPII-treated PQDs. (a) Current density-voltage curve, (b) Radiance-voltage curve, (c) EQE-radiance curve, and (d) shows the image of a lighted device.

1. **References**

[1] Wijaya, H. et al. Efficient near-infrared light-emitting diodes based on In(Zn)As–In(Zn)P–GaP–ZnS quantum dots. *Adv. Funct. Mater.* **30**, 1906483 (2020).

[2] De Franco, M. et al. Near-infrared light-emitting diodes based on RoHS-compliant InAs/ZnSe colloidal quantum dots. *ACS Energy Lett.* **7**, 3788-3790 (2022).

[3] Roshan, H. et al. Near infrared light-emitting diodes based on colloidal InAs/ZnSe core/thick-shell quantum dots. *Adv. Sci.* **11**, 2400734 (2024).

[4] Lim, L. J. et al. Non-toxic CuInS_2_/ZnS colloidal quantum dots for near-infrared light-emitting diodes. *Adv. Mater.* **35**, 2301887 (2023).

[5] Gao, L. et al. Efficient near-infrared light-emitting diodes based on quantum dots in layered perovskite. *Nat. Photonics* **14**, 227-233 (2020).

[6] Cheng, K. Y. et al. High-efficiency silicon nanocrystal light-emitting devices. *Nano Lett.* **11**, 1952-1956 (2011).

[7] Lignos, I. et al. Exploration of near-infrared-emissive colloidal multinary lead halide perovskite nanocrystals using an automated microfluidic platform. *ACS Nano* **12**, 5504-5517 (2018).

[8] Begum, R. et al. Stable Sn^2+^ doped FAPbI_3_ nanocrystals for near-infrared LEDs. *Chem. Commun.* **55**, 5451-5454 (2019).

[9] Chen, L. C. et al. A novel approach utilizing magnesium acetate as the Mg^2+^ doping source for enhanced stability and efficiency in FAPbI_3_ perovskite quantum dot LEDs. *Mater.* *Sci. Semicond. Process.* **189**, 109277 (2025).

[10] Abe, H. et al. Boosting the stability of FAPbI_3_ perovskite nanocrystal near-infrared light-emitting diodes with aromatic ligands and organic host dispersion. *Small* **21**, 2501159 (2025).

[11] Wang, Y. et al. Ligand-solvent coordination enables comprehensive trap passivation for efficient near-infrared quantum dot light-emitting diodes. *Angew. Chem. Int. Ed.* **63**, e202407833 (2024).

[12] Li, H. H. et al. Hybrid-dimensional heterostructure enables efficient near-infrared perovskite quantum dot light-emitting diodes. *ACS Nano* **19**, 25930-25938 (2025).

[13] Liu, Z. S. et al. Liquid bidentate ligand for full ligand coverage towards efficient near-infrared perovskite quantum dot LEDs. *Light Sci. Appl.* **14**, 35 (2025).
